# Cost profiles of colorectal cancer patients in Italy based on individual patterns of care

**DOI:** 10.1186/1471-2407-13-329

**Published:** 2013-07-05

**Authors:** Silvia Francisci, Stefano Guzzinati, Maura Mezzetti, Emanuele Crocetti, Francesco Giusti, Guido Miccinesi, Eugenio Paci, Catia Angiolini, Anna Gigli

**Affiliations:** 1 Centro Nazionale di Epidemiologia, Sorveglianza e Promozione della Salute, Istituto Superiore di Sanità, Roma, Italy; 2 Registro Tumori del Veneto, Istituto Oncologico Veneto-IRCCS, Padova, Italy; 3 Dipartimento SEFeMEQ, Università“Tor Vergata”, Roma, Italy; 4 Registro Tumori della Regione Toscana-ISPO, Firenze, Italy; 5 Dipartimento di Oncologia, ASF, Firenze, Italy; 6 Istituto di Ricerche sulla Popolazione e le Politiche Sociali, Consiglio Nazionale delle Ricerche, via Palestro 32, 00185, Roma, Italy

**Keywords:** Cancer registry, Prevalence, Administrative data, Direct costs, Care pathway

## Abstract

**Background:**

Due to changes in cancer-related risk factors, improvements in diagnostic procedures and treatments, and the aging of the population, in most developed countries cancer accounts for an increasing proportion of health care expenditures. The analysis of cancer-related costs is a topic of several economic and epidemiological studies and represents a research area of great interest to public health planners and policy makers. In Italy studies are limited either to some specific types of expenditures or to specific groups of cancer patients. Aim of the paper is to estimate the distribution of cancer survivors and associated health care expenditures according to a disease pathway which identifies three clinically relevant phases: initial (one year following diagnosis), continuing (between initial and final) and final (one year before death).

**Methods:**

The methodology proposed is based on the reconstruction of patterns of care at individual level by combining different data sources, surveillance data and administrative data, in areas covered by cancer registration.

**Results:**

A total colorectal cancer-related expenditure of 77.8 million Euros for 18012 patients (corresponding to about 4300 Euros per capita) is estimated in 2006 in two Italian areas located in Tuscany and Veneto regions, respectively. Cost of care varies according to the care pathway: 11% of patients were in the initial phase, and consumed 34% of total expenditure; patients in the final (6%) and in the continuing (83%) phase consumed 23% and 43% of the budget, respectively. There is an association between patterns of care/costs and patients characteristics such as stage and age at diagnosis.

**Conclusions:**

This paper represents the first attempt to attribute health care expenditures in Italy to specific phases of disease, according to varying treatment approaches, surveillance strategies and management of relapses, palliative care. The association between stage at diagnosis, profile of therapies and costs supports the idea that primary prevention and early detection play an important role in a public health perspective. Results from this pilot study encourage the use of such analyses in a public health perspective, to increase understanding of patient outcomes and economic consequences of differences in policies related to cancer screening, treatment, and programs of care.

## Background

In most developed countries, cancer is responsible for an increasing amount of national health expenditures [1]. The number of newly diagnosed cancer patients is expected to increase due to population growth and aging [2]. Furthermore, improvements have been achieved in reducing cancer mortality via prevention, early detection and effective new therapies, as a consequence the amount of people living with a cancer is increasing. Health care delivery trends, including increasing costs of cancer care and, in particular, increasing use of expensive new chemotherapy drugs [3,4] are projected to be associated with increased costs of cancer care. In this context, quantification of cancer costs is paramount in order to measure the economic burden of the disease and to predict the impact of new medical interventions [5].

The analysis of cancer-related costs is a topic of several epidemiological and economic studies. Some are based on the number of newly diagnosed cases (incident cases), others are based on survivors at a given calendar time (prevalent cases) [6]. In the prevalence-based approach patients are distributed into different disease phases according to the disease pathway and according to different care needs [7,8]. The cancer pathway is usually subdivided into three phases representing clinical and cost-related dynamic: initial (the time following diagnosis, usually one year after diagnosis), continuing (all time occurring between initial and final) and final (the time before death, usually one year before death). Most studies on patterns of care and costs of cancer have been conducted in countries where cancer registration is nation- or region-wide and prevalence can be estimated as a function of new cases and life status follow-up [9]. In these cases, data collected on diagnosis and life status of all incident cases give the most reliable basis for estimating prevalence as all new cases occurring in the population covered are registered. Health expenditure data are available both on individual level and for different cancer sites for specific sub-groups of population (as is the case of the Medicare database, which includes only patients of 65 and over [10]).

In Italy in year 2006 2.2 million of cancer survivors have been estimated [11]. The total health expenditures have been quantified as 110 billion euros (7.3% of Gross Domestic Product) and expenditures attributable to cancer as 7.5 billion euros, 6.7% of total health expenditures [12]. In Italy there is local experience limited either to some specific types of expenditures or to specific disease phases. Moreover some experience exists on cost-effectiveness analyses, aimed to evaluate specific cancer screening programs finalized to early detection of cases [13-19]. These studies are based on macroeconomic data related to specific procedures or screening interventions.

In this work we aim to: a) estimate the distribution by phase of care of prevalent cases of colon and rectal cancer patients in two Italian areas in 2006, and b) estimate total direct expenditures sustained by the public health care system to provide hospital care for those colorectal cancer survivors for one year, given the age class, the stage at diagnosis and the phase of care, by using information on individual patient pathways of hospital care (from diagnosis to possible recovery or death). The paper describes a pilot study innovative with respect to the previous experiences in the Italian context because it allows to identify subgroups of cancer survivors homogeneous with respect to their health care needs and the estimation of the corresponding economic resources allocated to each subgroup during hospitalization. In order to apply the methodology proposed here, we need to combine two sources of information: a surveillance source, containing individual level clinical information on the patient disease; an administrative source, containing individual level information on the procedures and interventions undergone by the patient during hospitalization.

## Data and methods

Data needed to estimate the cost profiles and the cancer survivors are from two different sources: population-based Cancer Registries (CR) and Hospital Discharge Cards database (HDC). Data is provided by cancer registries. The Italian legislation identifies Cancer Registries as collectors of personal data for surveillance purposes without explicit individual consent. The approval of a research ethic committee is not required, since this study is a descriptive analysis of individual data without any direct or indirect intervention on patients.

### Cancer registry database

Population-based cancer registries collect data on all cancer diagnoses occurring in the population resident in the area covered by the cancer registration. Patients registered are then actively followed up with respect to their vital status, using the information from the National Death Certificate Database. In Italy cancer registries cover about 34% of the population and are located mostly in Northern and Central areas of Italy. In this study the cancer registries of Veneto and Tuscany [20] are involved. Veneto Cancer Registry (VCR) covers about 1.8 millions inhabitants resident in the North-Eastern region of Veneto, representing 38% of the whole region [21]. VCR database contains all cases diagnosed with cancer from 1990 to December 31, 2005 and followed up to December 31, 2007. Tuscany Cancer Registry (TCR) covers the population resident in the two provinces of Firenze and Prato (1.2 million residents), representing 33% of the whole Tuscany region, located in Central Italy [22]. TCR database includes all cases diagnosed with cancer from 1985 to December 31, 2005 and followed up to December 31, 2007. For each patient the following information is available in the cancer registry: date of birth, date of diagnosis, sex, vital status, tumor site, morphology, diagnostic confirmation. Data from VCR and TCR are used to compute the Limited Duration Prevalence [23], i.e. the number of registered patients diagnosed with colorectal cancer and still alive at prevalence date and the Complete Prevalence [24], i.e. the number of survivors at prevalence date who had a colorectal cancer as first primary diagnosis in their life.

### Hospital discharge card database

In Italy a public welfare system guarantees universal health care. The national health service is centrally organized under the Ministry of Health and it is administered on a regional basis (19 regions and 2 provinces).

Hospitals are reimbursed by the regional governments according to the Diagnosis-related group (DRG) system [25], whereby they receive a lump sum payment for each patient, determined by the patient’s diagnosis, health status, and procedures performed during the hospitalization. For each hospital admission a HDC is filled by the doctors who take care of the patient. A HDC refers to a single hospital admission by a single individual and contains demographic information (date of birth, sex, place of birth, place of residence), clinical information (type of diagnosis, interventions and procedures coded by the ICD9-CM classification [26]), and administrative information (coded by the DRG coding system). Different HDCs related to the same individual can be traced thanks to a personal identification code or to a number of information related to the patient (last name, first name, gender, date of birth, place of birth).

### Study population

Two incident cohorts of colorectal cancer patients diagnosed during the period January 1, 2000-January 1, 2002 in the TCR area and in the Local Health Unit (LHU) of Padua (381.000 inhabitants, about 20% of the entire VCR area) are considered for linkage to the HDC, in order to estimate the cost profiles. For these patients information on stage at diagnosis classified according to the TNM staging system [27] is also provided, and 15% of cases with unknown stage (subdivided almost equally between those who underwent surgery and those who did not) are excluded from the estimation of cost profiles. The main features of the two cohorts are summarized in Table 1 the total number of patients is 2060 for TCR and 607 for LHU-Padua, with men and women almost equally represented, and a similar age structure. Differences between TCR and LHU-Padua, stage distributions were tested and are statistically significant: with more Stage I patients in LHU-Padua compared to TCR and vice versa for Stage II patients. These differences are possibly due to the cancer registry attitude in the stage classification and disappear when combining the two stages.

**Table 1:** Description of the TCR and LHU-Padua incidence cohorts 2000-2001

		**TCR**		**LHU-Padua**	
		**(N = 2060)**		**(N = 607)**	
		**N**	**%**	**N**	**%**
Gender					
	Male	1135	55.1	356	58.6
	Female	925	44.9	251	41.4
Age					
	15–69	916	44.5	294	48.4
	70–79	694	33.7	207	34.1
	80–99	450	21.8	106	17.5
TNM stage					
	I	262	12.7	106	17.5
	II	556	27.0	118	19.4
	III	537	26.1	162	26.7
	IV	397	19.3	135	22.2
	unstaged with surgery	166	8.0	45	7.4
	unstaged without surgery	142	6.9	41	6.8
Vital status at Dec.31, 2007					
	Alive	883	42.9	286	47.1
	Deaths due to colorectal cancer	892	43.3	224	36.9
	Deaths due to other cancer	105	5.1	39	6.4
	Deaths due to cardiovascular diseases	97	4.7	24	4.0
	Deaths due to other causes	83	4.0	34	5.6
Relative survival (age-adjusted)					
	Years	%	s.d.	%	s.d.
	1	82.0	0.91	85.8	1.5
	2	73.0	1.10	77.5	1.86
	5	60.0	1.33	64.8	2.31

There is a statistically significant difference between the relative survival (age-adjusted values according to Corazziari standard population [28]) of the two cohorts: LHU-Padua has higher relative survival than TCR during the entire follow-up span. The main reason for these differences is the case-mix of patients with missing stage with respect to survival.

Each case from the two incident cohorts is linked to the HDC database, in order to trace all hospital discharges referred to the patient, starting from his/her diagnosis up to January 1, 2007. HDC reporting codes of diagnoses (Additional file 1: Table S1), interventions and procedures related to colorectal cancer (Additional file 1: Table S2) and used in the two regions since the beginning of 2000 are taken into account.

The linkage is deterministic and 95% of all colorectal cancer cases are linked to one or more HDC: a total number of 607 patients were linked with 3853 HDC in LHU-Padua, and a total number of 2060 patients were linked with 7896 HDC in TCR. Incident cases not linked with the HDC database are: those diagnosed with Death Certificate Only (DCO) or discovered at autopsy; cancer patients who are diagnosed and whose cancer is treated in outpatient clinic (i.e. outside of the hospital) or in private hospitals not included in the National Health System.

### Estimation of costs and cost profiles

Reimbursements from the regional government corresponding to individual DRG codes (details in Additional file 1: Table S3) are used to derive single patient cost profiles.

Each patient in the cohort spends a varied number of months in each phase of the disease (initial, continuing, final) and has a different disease pathway (with one phase only to three phases) according to the following criteria:

• diagnosed and dead within 12 months: such patient may contribute to hospital costs only in the final phase;

• diagnosed and dead between 13 and 24 months: such patient may contribute to hospital costs only in the initial and final phase of the disease; the final phase has priority over the initial phase;

• diagnosed and alive after 24 months, dead any time before January 1, 2007: such patient may contribute to hospital costs in the initial, continuing, and final phase of the disease;

• diagnosed and still alive at January 1, 2007: such patient may contribute to hospital costs in the initial and continuing phase of the disease;

In each disease phase monthly average costs are obtained by dividing the total costs of the phase by the total number of person-months in the same phase (computational details in Appendix). Notice that the contribution may be either in terms of person-months and costs or of person-months only: a patient still alive at prevalence date contributes to the denominator even if s/he has not being hospitalized and has no hospital costs. Average costs on yearly basis are obtained by multiplying the monthly average costs by 12 for each phase of care.

The set of the values corresponding to the three phases of care constitutes the yearly cost profile.

In our case HDC collected in the period January 1, 2000 to January 1, 2007 refer to patients diagnosed in 2000-2001, hence initial phase costs are averaged over the period 2000-2002, continuing phase Table 1 costs are averaged over the period 2001-2006, and final phase costs are averaged over the period 2000-2006.

Yearly cost profiles are specific by age class (<70; 70-79; 80+), chosen according to clinical characteristics and clinicians attitudes toward treatment options, and in the initial phase are also specific by stage at diagnosis. Yearly cost profiles derived for the LHU-Padua are used for the entire VCR area, assuming a common reimbursement distribution by phase of care (VCR cost profiles).

### Statistical methods: prevalence decomposition

Suppose a cancer registry has length of registration of K years at the beginning of year Y and information on vital status is available up to year (Y + 1). We are interested in decomposing the observed prevalent cases into three components, given by phases of care:

• P^1^_Y_ are the prevalent cases in the initial phase, that is cases observed in Y who were diagnosed within the previous 12 months;

• P^2^_Y_ are the prevalent cases in the continuing phase, that is cases observed in Y who were diagnosed more than 12 months before and are alive at the beginning of year Y + 1 ;

• P^3^_Y_ are the prevalent cases in the final phase, that is cases observed alive in Y who were diagnosed any time after the initial year (Y-K) and died between Y and Y + 1.

Prevalent cases in each phase are calculated by age class (for simplicity we omit the age index) according to the number of years since diagnosis as follows: let LDP_Y_ denote the Limited Duration Prevalence at January 1, year Y and let LDP_Y_(j) be the number of prevalent cases in Y who were diagnosed during the year Y-j , with j = 1,…,K; these cases have survived j years, therefore have duration j; both LDP_Y_ and LDP_Y_(j) are obtained from the SEER*Stat software [29] and are adjusted to take into account lost-to-follow up cases. Similarly let D_Y_(j) be the number of prevalent cases who die in year Y and were diagnosed during the year Y-j, as reported from the National Death Certificate Database.

In the implementation to VCR and TCR data, the distribution of prevalent cases by phase of care has been obtained using prevalent cases at January 1, 2006 and number of deaths occurred from January 1, 2006 to January 1, 2007, according to the following criteria:

1) The number of prevalent cases in the initial phase are those cases diagnosed during the year 2005 (incident cohort 2005 diagnosed from January 1 to December 31) who will survive until January 1, 2007. Prevalent cases in the initial phase have been further stratified by stage at diagnosis, using the 2000-2002 incidence cohort stage distribution.

2) The number of prevalent cases in the continuing phase are survivors at January 1, 2006 of duration j in [2,K] still alive at January 1, 2007. Cases diagnosed before the registry started its activity and still alive, can only be in the continuing or final phase; here we assume that they are in the continuing phase. The reasoning behind this assumption is that for Cancer Registries with long length of activity, it is likely that cases diagnosed before the cancer registration started and still alive in Y are cured and will never die for the first primary tumor they were diagnosed with. More specifically, when registry length K is higher than the mean survival time for fatal cases (those who are bound to die for the cancer considered), survivors after K years may be considered as cured and can be entirely attributed to the continuing phase. Previous studies showed that for colon and rectum cancers the proportion of cured patients in Italy is about 46% and the mean survival time for fatal cases is about two years and a half [30]. We therefore expect that the unobserved prevalent cases in TCR and VCR in 2006 are all cured and belong to the continuing phase of the disease.

3) Finally, the number of prevalent cases in the final phase are those who will die during the year 2006.

## Results and discussion

**Figure 1:**
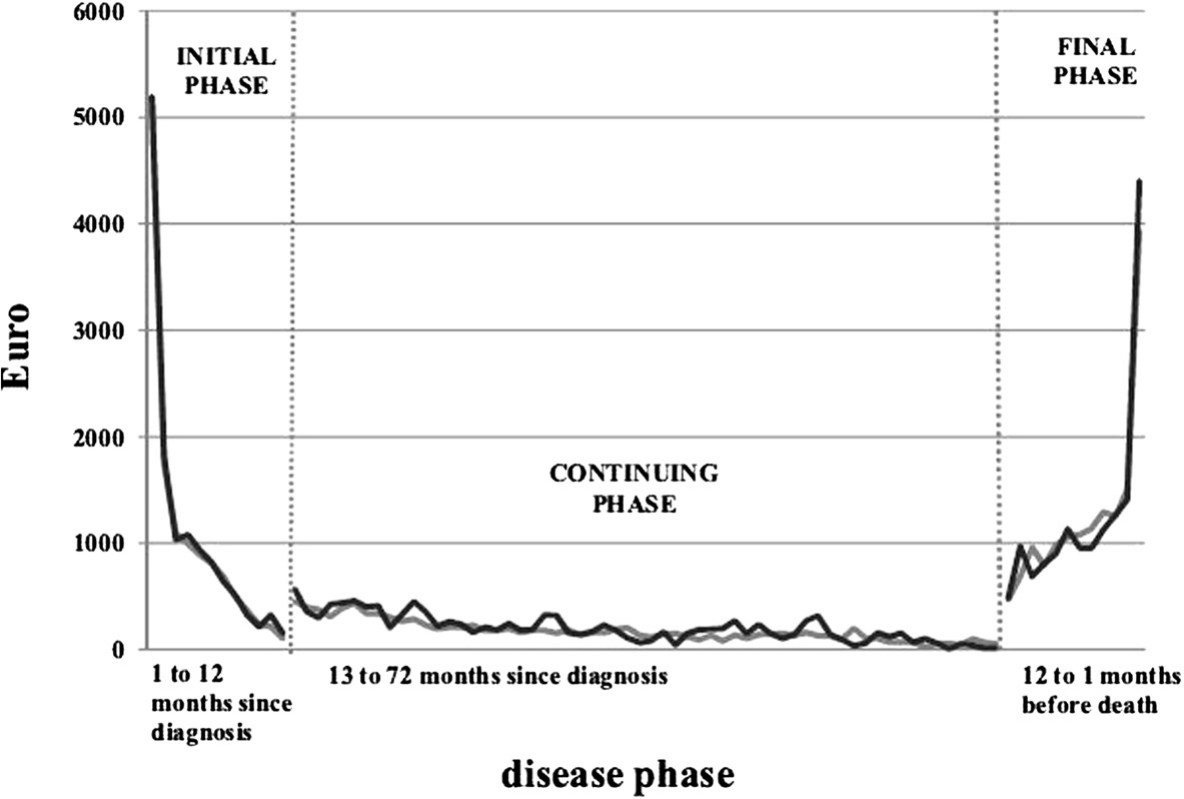
**Average costs (€) per month of colorectal cancer patients by phase of care.** The figure shows the dynamic along the disease pathway of the average reimbursement (in Euros) allocated to hospitals per patient/per month (y-axis) in each phase of the disease of the 2000-2001 cohort of colorectal cancer cases diagnosed in LHU-Padua and TCR respectively. In the initial and continuing phases the x-axis measures the time occurred since diagnosis, in the final phase the x-axis measures the time since entrance in the final phase (starting from the twelfth up to the first month since entrance). The black line represents the LHU-Padua, the gray line represents the TCR.

Figure 1 shows the average costs per month by phase of care in LHU-Padua and TCR, for all patients combined, regardless of their distribution by age and stage at diagnosis. The costs in the figure are expressed in Euros and represent the average reimbursement allocated to hospitals per patient/per month in each phase of the disease. In the initial and continuing phases the horizontal axis measures the time occurred since diagnosis, in the final phase the horizontal axis measures the time since entrance in the final phase (starting from the twelfth up to the first month since entrance). Notice that by computing monthly averages, different survival patterns within the same disease phase are taken into account, because individuals contribute to costs (numerator) and cases (denominator) only for the actual number of months they are in a specific phase. The results show a U-shaped cost profile, with highest costs in the initial and final phases of care and lower costs in the continuing phase of care.

The cost profile shows very high costs during the first months of the initial phase (about 5000 € per person/month), then declining until reaching a plateau during the continuing phase (about 200 € per person/month on average) and then increasing again during the last phase of the disease. The peak during the last months of life at the end of the U-shape is caused by the contribution of short survivors (i.e. those patients who survive less than three month), these patients have a monthly average cost of about 4 000 € in the last month of life and about 1 500 € in the second and third months before death. Consistently with the literature [31] these cases are allocated to the final phase, however their treatments are a mixture of diagnostic and surgical procedures, which are more expensive, and terminal care. If we did not consider these cases (which represent 18% in LHU-Padua and 23% in TCR) the costs of the final phase would be gradually increasing, without reaching a peak.

**Figure 2:**
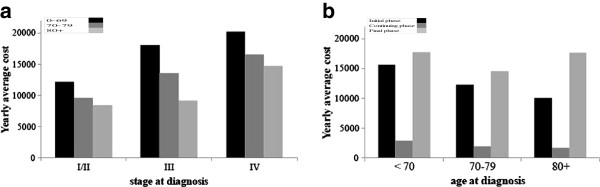
**Average yearly costs (€) of colorectal cancer patients for VCR and TCR combined. a**. The histogram illustrates the distribution of the average yearly costs (in Euros) of the initial phase in the 2000-2001 cohort of colorectal cancer cases diagnosed in VCR and TCR combined, by stage at diagnosis (x-axis) and age class (dark bar represents 0-69, gray bar represents 70-79, light gray bar represents 80+). **b**. The histogram illustrates the distribution of the average yearly costs (in Euros) in the 2000-2001 cohort of colorectal cancer cases diagnosed in VCR and TCR combined, by age at diagnosis (x-axis) and phase of care (dark bar represents the initial phase, gray bar represents the continuing phase, light gray bar represents the final phase).

Figure 2 show the yearly cost profiles for LHU-Padua and TCR combined. In Figure 2a each bar represents the average reimbursement (in Euros) allocated to hospitals for all cancer-related treatments and procedures provided to a patient in the initial phase by stage at diagnosis (X axis) and age class (different color of the bars). In Figure 2b each bar represents the average reimbursement (in Euros) allocated to hospitals for all cancer-related treatments and procedures provided to a patient by age class (X axis) and phase of care (different color of the bars).

There is a trend in costs by stage at diagnosis in the initial phase in all age classes: more advanced stages correspond to higher average costs, patients in stage IV cost about 70% more than patients in early stages I and II (Figure 2a). This result is consistent with the clinical guidelines and recommendations, which suggest different treatment strategies according to the tumor stage: typically, patients with tumor in Stage I are followed up without adjuvant therapy, while for a portion of high risk Stage II patients (about 20%) adjuvant therapy after surgery could be considered [32,33]; Stage III patients are treated with a curative surgical resection (possibly with transient colic anastomosis) and postoperative chemotherapy is mandatory; standard treatments for Stage IV patients have primarily palliative intent, and consist of surgical resection of bowel and/or metastasis, palliative anastomosis, chemotherapy (possibly in association with biological therapy) and radiation therapy to the primary rectal tumor to palliate bleeding, or to disease metastasis to palliate pain [34,35]. In this analysis, the decision to combine Stages I and II, in order to obtain more robust estimates comparable between the two cancer registries, is justified by the similarity between cost profiles observed in Stage I and Stage II separately (data not shown).

Age is also related with costs: younger patients entail from 40% to 100% more expenditures than oldest patients (80+) in all phases of the disease. There is a large debate about the impact of patients age on the decision making: despite the presence of comorbidities among elderly patients, age itself might not be a predictor of outcome, and the efficacy of adjuvant treatments as well as the toxicity of chemotherapy are similar to the general population [36]. The U-shape of costs by phase of care is confirmed for all age classes (Figure 2b), as indicated in the literature [37]: with 10 to 17 thousands Euros yearly costs on average for the initial and final phases, 1.7 to 2.9 thousands Euros yearly costs on average for the continuing phase.

Table 2 illustrates the number of colorectal cancer survivors (prevalent cases) in 2006 by age class and phase of care in TCR and VCR respectively, and the corresponding yearly costs in 2006, expressed in Euros. The picture of the situation is similar in VCR and TCR, with most prevalent cases and economic resources concentrated in the continuing phase, followed by lower proportion of cases and costs in the initial phase and a minority of final cases using about 1/4 of the budget. A total expenditure of 44.5 million euros for 9640 patients is estimated for VCR, while 33.3 million euros for 8357 patients is for TCR. These figures correspond to about 4300 euros per capita in 2006. The differences between patterns of prevalent cases and patterns of costs by phase of care are due to the effect of average costs of care which varies according to the disease and care pathway: 83% of cases in the continuing phase correspond to 43% of health expenditure, mainly devoted to monitoring procedures, which are less costly; 11% of patients in the initial phase use about 34% of the total budget for diagnostic and surgical procedures, that are more costly; final phase includes only 6% of cases but requires about 23% of the total budget. The consistency of the distributions of prevalent cases and costs between the two registries is a relevant result, being a pre-requirement for extrapolation of the methodology at regional level.

**Table 2:** TCR and VCR prevalent cases at Jan. 1, 2006 and costs (in Euros) by phase care and age class

		** TCR**				** VCR**			
**Phase of care**	**Age class**		**Prevalent cases**		**Costs (Euros)**		**Prevalent cases**		**Costs (Euros)**
**Initial**	15-69		373		5 778 956		556		9 302 077
	70-79		268		3 371 999		342		4 093 306
	80-99		190		1 701 665		203		2 445 414
Subtotal (%)	*All ages (10*%*)*		*831 (32*%*)*		*10 852 620 (11*%*)*		*1101 (36*%*)*		*15 840 797*
**Continuing**	15-69		2331		6 45 7 952		2952		9 101 016
	70-79		2411		4 187 039		2792		6 053 056
	80-99		2306		3 827 130		2214		4 004 222
Subtotal (%)	*All ages (84*%*)*		*7048(43*%*)*		*14 472 121(83*%*)*		*7958 (43*%*)*		*19 158 294*
**Final**	15-69		106		1 337 504		156		2 731 404
	70-79		134		1 975 533		163		2 339 350
	80-99		251		4 650 260		264		4 447 608
Subtotal (%)	*All ages (6*%*)*		*491(25*%*)*		*7 963 297 (6*%*)*		*583 (21*%*)*		*9 518 362*
**Total**			**8370**		**33 288 038**		**9642**		**44 517 453**

## Conclusions

This paper describes the colorectal cancer burden in 2006 in two Italian areas covered by cancer registration starting from individual patterns of hospital care. The idea is to provide the distribution of health care expenditures according to the disease pathway from first diagnosis to possible recovery or death, for subgroups of cancer survivors homogeneous with respect to their health care needs.

The phase of care approach here used subdivides care into three clinically relevant intervals: the first year since diagnosis, the last year of life and the monitoring or continuing period. The main advantage of the phase of care approach is the possibility to describe the distribution by disease phase of prevalent cases and hospital care expenditures at a given date taking into account the individual patterns of care during the entire lifespan. The methodology requires the registration of new cancer cases and follow-up information, typically provided by the cancer registries, and the collection of data on treatments and corresponding costs, derived from other administrative sources. To our knowledge this represents the first experience in Italy in estimating prevalent cases and costs distribution according to a three- phase of care framework and linking, at individual level, CR information to the HDC database.

Data linkage is the preliminary step in order to reconstruct the patterns of care along the entire disease pathway. Clinical guidelines are insufficient to predict patterns of care and furthermore compliance with clinical guidelines might vary between geographical areas. Following the individual record linkage, DRG codes have been accurately selected in order to identify only colorectal cancer-related treatments, i.e. those treatments appropriately attributable to the colon and rectum primary. One of the advantages of this procedure is that it allows the direct estimation of the net cancer costs, without using a control cohort of non-cancer subjects matched to patients by sex, age, location area and phase of care, as elsewhere proposed in the literature [10]. Another advantage is the direct identification of prevalent cases in the final phase by counting the number of deaths during the following year – information on life status follow-up is provided by the CR using the National Death Certificate Database– rather than estimating a survival curve, as elsewhere proposed [8].

Stage is a key variable in this study, because it determines the treatment approach and the corresponding patterns of care and costs. Stage classification is a complex issue, and different attitudes in the definition of stage at diagnosis might compromise the comparability of data between cancer registries. In our study, however, stage at diagnosis is used merely as a proxy of treatment approach and an accurate stage classification is not required.

In the disease phase framework the number of phases and length of each phase are a-priori defined and do not vary according to cancer site or patients characteristics; however, a twelve months initial phase might be too short compared to the time required to complete some first course treatments [38]; the continuing phase length may vary according to patient survival and corresponding care needs depend on additional prognosis factors, such as the presence of metastases and possible comorbidities, which are not taken into account in the analysis; a flexible estimation of the phase number and duration based on observed disease pathways of cancer patients cohorts might therefore be envisaged [39].

Short term survivors are attributed to the final phase only, regardless of their cause of death and the fact that they might have received first course procedures and treatments. This might lead to a possible overestimation of the number of terminal patients and of the total costs attributed to final phase. A correction factor based on the observed proportion of other causes of death in the incident cohorts could be implemented.

Costs of the initial phase refer to a cohort of patients diagnosed in 2000-2001 and do not include recent variations in the cost of drugs and new diagnostic procedures and treatments. Relevant changes in first course therapies of colorectal cancer patients have been occurred in Italy after 2005. As a further development other sources of data in addition to in-hospital records should be included, i.e. data on drugs consumption and outpatient treatments, in order to obtain a complete estimation of costs directly attributable to a specific cancer.

In Italy CR’s represent a reliable surveillance source which however covers a third of the national population and it is not representative of the whole country; furthermore, most of the areas covered by the CRs correspond only to portions of a region; finally, each region is an autonomous entity regarding health care administration. As a consequence of these features, extrapolating costs at national level is a very complex exercise. On the other hand extrapolation at regional level is in principle feasible, but requires some further methodological steps, such as projecting prevalent cases in areas not covered by CR’s, which goes beyond the scope of this pilot study, and will be the goal of a future development.

A validation of our cost estimates using comparable figures of health care expenditures documented in the regional budget plans of Veneto [40] and Tuscany [41] has been carried out: a total health expenditure of 44.5 million Euros and 33.8 million Euros, in VCR and TCR respectively, is estimated for 9640 and 8357 colorectal prevalent cases respectively in 2006, and the distribution of costs by phase of care is similar between the two cancer registries; the total costs per capita of colorectal cancer patients, regardless the phase of care, is about 4 300 Euros per year in the two cancer registries combined, and this value is consistent with a total health expenditures of about 5 000 reported in the regional budget 2008. The comparability of the results between the two CR areas confirms the consistency with the clinical guidelines and represents a first step for future analyses with the aim to extrapolate local results to the entire regions.

Finally, the methodology proposed and applied in this paper may be improved with a projection tool in order to evaluate the impact of specific public health interventions on patterns of care and costs of cancer patients. The analysis of the effects of cancer control strategies, such as screening programs or new treatments introduction, on the expected economic burden will be an important area for additional research in a public health perspective.

## Appendix

### Estimation of average costs by phase of care

Let X be a sample of patients for which we want to estimate the hospital costs and the profile of costs according to phase of care f (= 1,…,3), age group a (= 1,…,3), and stage at diagnosis s (= 1,…,3), and let X_a,s_ be the subgroup of same age and stage at diagnosis.

The cost profile is a 3-dimensional vector

$${\bar{C}}_{a,s}=\left(c_{a,s}^{1},c_{a}^{2},c_{a}^{3}\right)$$

where the components represent the yearly average costs of each phase, and are computed as follows:let c^f^_ij_ be the costs of patient j (X_a,s_) in month i (=1,…,M^f^) of phase f, and let s^f^_ij_ denote an indicator of the contribution of patient j in month i of phase f: s^f^_ij_ is equal to 1 if patient j is alive in month i of phase f, and equal to 0 otherwise. In order to compute the yearly average costs of each phase we must divide the total costs of the phase by the total number of person-months in the same phase. Total costs are obtained aggregating the hospitalization costs of each patient observed in each month associated to the phase.

In our case we observe

M^1^ =12 months in phase 1, the initial phase may start any time between Jan 1, 2000 and Jan 1, 2002 and lasts a number of months variable for each patient between 0 and 12;

M^2^ = 72 months in phase 2, because the continuing phase may start any time between Jan 1, 2001 and Jan 1, 2006, and lasts a number of months variable for each patient between 0 and 72 months (up to the maximum follow-up 01/01/2007);

M^3^ = 12 months in phase 3, because the final phase may occur any time between Jan 1, 2000 and Jan 1, 2007, and lasts a number of months variable for each patient between 1 and 12 months.

Furthermore the variable stage at diagnosis only affects treatments, and consequently costs, in the initial phase, and will not appear in the continuing and final phase.

The yearly average costs are

$${\bar{c}}_{a,s}^{1}=\frac{\sum_{j\in X_{a,s}}\sum_{i=1}^{M^{1}}c_{\mathrm{ij}}^{1}}{\sum_{j\in X_{a,s}}\sum_{i=1}^{M^{1}}s_{\mathrm{ij}}^{1}}12,$$

and

$${\bar{c}}_{a}^{f}=\frac{\sum_{j\in X_{a}}\sum_{i=1}^{M^{f}}c_{\mathrm{ij}}^{f}}{\sum_{j\in X_{a}}\sum_{i=1}^{M^{f}}s_{\mathrm{ij}}^{f}}12,$$

for f = 2,3.

### Prevalence decomposition

Let Y indicate the year and K the number of years a registry has been in activity; let LDP_Y_ denote the Limited Duration Prevalence at year Y (January 1st) , and let LDP_Y_(j) be the number of prevalent cases in Y who were diagnosed during year Y-j (j = 1,…,K); these cases have survived j years, therefore have duration j. Similarly let D_Y_(j) be the number of prevalent cases who die during year Y and were diagnosed during year Y-j. We want to decompose the prevalent cases according to the three phases of care: initial, continuing, final.

In our context the number of prevalent cases in the initial phase are those cases diagnosed during the year 2005 (incident cohort 2005 diagnosed from January 1 to December 31) who will survive until January 1, 2007. These cases are derived by subtracting from the number of cases alive at January 1, 2006 (prevalent cases 2006 of duration 1: LDP_2006_(1)) the number of deaths occurred in the incident cohort 2005 during the year 2006, computed as the average between the number of deaths occurred in 2006 of duration 1 (D_2006_(1)) and the number of deaths occurred in 2006 of duration 2 (D_2006_(2)):

$$P_{2006}^{1}=\mathrm{LD}P_{2006}\left(1\right)-\frac{D_{2006}\left(1\right)+D_{2006}\left(2\right)}{2},$$

where duration 1 corresponds to the time interval [0,1), and duration 2 corresponds to the time interval [1,2).

The number of prevalent cases in the continuing phase are survivors at January 1, 2006 of duration j in [2,K] still alive at January 1, 2007. These cases are derived by subtracting from the number of prevalent cases at January 1, 2006 of duration j, the average number of deaths occurred in 2006 of durations j and j + 1 and summing up these differences for each duration:

$$\sum_{j=2}^{k}\left(\mathrm{LD}P_{2006}\left(j\right)‒\frac{D_{2006}\left(j\right)+D_{2006}\left(j+1\right)}{2}\right)$$

Furthermore, we add to the continuing phase cases diagnosed before the registry started its activity and still alive, P^U^_2006_ , and obtain

$$P_{2006}^{2}=\sum_{j=2}^{k}\left(\mathrm{LD}P_{2006}\left(j\right)‒\frac{D_{2006}\left(j\right)+D_{2006}\left(j+1\right)}{2}\right)+P_{2006}^{U},$$

where P^U^_2006_ is obtained via the complete prevalence method and computed in the COMPREV software [42].

Finally, the number of prevalent cases in the final phase are those who will die during the year 2006. These cases are derived by summing up all average numbers of deaths occurred during the year 2006 in each duration j in [1,K]:

$$P_{2006}^{3}=\sum_{j=1}^{K}\frac{D_{2006}\left(j\right)+D_{2006}\left(j+1\right)}{2}.$$

## Abbreviations

CR: Cancer registry; DCO: Death certificate only; DRG: Diagnosis-related group; HDC: Hospital discharge card database; LDP: Limited duration prevalence; TCR: Tuscany cancer registry; VCR: Veneto cancer registry.

## Competing interests

The authors declare that they have no competing interests.

## Authors’ contributions

SF, AG conceived the study, participated in its design and coordination and helped to draft the manuscript. MM conceived the study and participated in its design. SG participated in the design of the study, performed the statistical analysis and helped to draft the manuscript. FG participated in the design of the study and performed the statistical analysis. EC, GM, EP, CA participated in the design of the study and helped to draft the manuscript. All authors read and approved the final manuscript.

## Pre-publication history

The pre-publication history for this paper can be accessed here:

http://www.biomedcentral.com/1471-2407/13/329/prepub

## Supplementary Material

Additional file 1: Table S1 List of colon-rectum cancer-related hospital diagnoses. **Table S2.** List of colon-rectum cancer-related hospital procedures. **Table S3.** Table of the more frequent Diagnosis Related Group codes related to colorectal cancer incidence cohorts 2000-2001. The table contains the list of the most frequent DRG codes (overall representing about **80%** of all cases) associated to colorectal cancer incidence cohorts 2000-2001. For each DRG [43] description and related reimbursement rate (cost in Euros) are also reported.
Click here for file
