# Supplementary material for: Cost profiles of colorectal cancer patients in Italy based on individual patterns of care
Source: BMC Cancer. 2013 Jul 5;13:329. doi: 10.1186/1471-2407-13-329 (PMC3706387; doi:10.1186/1471-2407-13-329)
Supplement: Additional file 1: Table S1 — List of colon-rectum cancer-related hospital diagnoses. Table S2. List of colon-rectum cancer-related hospital procedures. Table S3. Table of the more frequent Diagnosis Related Group codes related to colorectal cancer incidence cohorts 2000-2001. The table contains the list of the most frequent DRG codes (overall representing about 80% of all cases) associated to colorectal cancer incidence cohorts 2000-2001. For each DRG [43] description and related reimbursement rate (cost in Euros) are also reported. [file 1471-2407-13-329-S1.docx]

Additional files

Table 1: List of colon-rectum cancer-related hospital diagnoses.

International Classification of Diseases 9th revision, version 2002. Effective in Italy since January 1, 2006

153 Malignant neoplasm of colon

154 Malignant neoplasm of rectum, rectosigmoid junction, and anus

158.8 Malignant neoplasm of specified parts of peritoneum

158.9 Malignant neoplasm of peritoneum, unspecified

159.0 Malignant neoplasm of intestinal tract, part unspecified

159.8 Malignant neoplasm of other sites of digestive system and intra-abdominal organs

159.9 Malignant neoplasm of ill-defined sites within the digestive organs and peritoneum

195.2 Malignant neoplasm of other and ill-defined sites of abdomen

197.5 Secondary malignant neoplasm of large intestine and rectum

197.7 Liver, specified as secondary

211.3 Benign neoplasm of colon

211.4 Benign neoplasm of rectum and anal canal

230.3 Carcinoma in situ of colon

230.4 Carcinoma in situ of rectum

230.5 Carcinoma in situ of anal canal

230.6 Carcinoma in situ of anus, unspecified

235.2 Neoplasms of uncertain behavior of stomach, intestines and rectum

560.9 Unspecified intestinal obstruction

569.2 Stenosis of rectum and anus

569.3 Hemorrhage of rectum and anus

V58.0 Radiotherapy

V58.1 Encounter for chemotherapy and immunotherapy for neoplastic conditions

V10.00 Personal history of malignant neoplasm of gastrointestinal tract, unspecified

V10.05 Personal history of malignant neoplasm of large intestine

V10.06 Personal history of malignant neoplasm of rectum, rectosigmoid junction, and anus

V44.2 Ileostomy status

V44.3 Colostomy status

V55.2 Attention to ileostomy

V55.3 Attention to colostomy

Table 2: List of colon-rectum cancer-related hospital procedures.

International Classification of Diseases 9th revision, version 2002. Effective in Italy since January 1, 2006

40.11 Biopsy of lymphatic structure

40.24 Excision of inguinal lymph node

45.2 Diagnostic procedures on large intestine

45.21 Transabdominal endoscopy of large intestine

45.22 Endoscopy of large intestine through artificial stoma

45.23 Colonoscopy

45.24 Flexible sigmoidoscopy

45.25 Closed [endoscopic] biopsy of large intestine

45.26 Open biopsy of large intestine

45.27 Intestinal biopsy, site unspecified

45.28 Other diagnostic procedures on large intestine

45.29 Other diagnostic procedures on intestine, site unspecified

45.4 Local excision or destruction of lesion or tissue of large intestine

45.41 Excision of lesion or tissue of large intestine

45.42 Endoscopic polypectomy of large intestine

45.43 Endoscopic destruction of other lesion or tissue of large intestine

45.49 Other destruction of lesion of large intestine

45.7 Open and other partial excision of large intestine

45.71 Open and other multiple segmental resection of large intestine

45.72 Open and other cecectomy

45.73 Open and other right hemicolectomy

45.74 Open and other resection of transverse colon

45.75 Open and other left hemicolectomy

45.76 Open and other sigmoidectomy

45.79 Other and unspecified partial excision of large intestine

45.8 Total intra-abdominal colectomy

45.9 Intestinal anastomosis

45.90 ntestinal anastomosis, not otherwise specified

45.91 Small-to-small intestinal anastomosis

45.92 Anastomosis of small intestine to rectal stump

45.93 Other small-to-large intestinal anastomosis

45.94 Large-to-large intestinal anastomosis

45.95 Anastomosis to anus

46.01 Exteriorization of small intestine

46.03 Exteriorization of large intestine

46.04 Resection of exteriorized segment of large intestine

46.1 Colostomy

46.10 Colostomy, not otherwise specified

46.11 Temporary colostomy

46.13 Permanent colostomy

46.14 Delayed opening of colostomy

46.2 Ileostomy

46.20 Ileostomy, not otherwise specified

46.21 Temporary ileostomy

46.22 Continent ileostomy

46.23 Other permanent ileostomy

46.24 Delayed opening of ileostomy

46.4 Revision of intestinal stoma

46.40 Revision of intestinal stoma, not otherwise specified

46.41 Revision of stoma of small intestine

46.42 Repair of pericolostomy hernia

46.43 Other revision of stoma of large intestine

46.5 Closure of intestinal stoma

46.50 Closure of intestinal stoma, not otherwise specified

46.51 Closure of stoma of small intestine

46.52 Closure of stoma of large intestine

46.93 Revision of anastomosis of small intestine

46.94 Revision of anastomosis of large intestine

48.2 Diagnostic procedures on rectum, rectosigmoid and perirectal tissue

48.21 Transabdominal proctosigmoidoscopy

48.22 Proctosigmoidoscopy through artificial stoma

48.23 Rigid proctosigmoidoscopy

48.24 Closed [endoscopic] biopsy of rectum

48.25 Open biopsy of rectum

48.26 Biopsy of perirectal tissue

48.29 Other diagnostic procedures on rectum, rectosigmoid and perirectal tissue

48.3 Local excision or destruction of lesion or tissue of rectum

48.31 Radical electrocoagulation of rectal lesion or tissue

48.32 Other electrocoagulation of rectal lesion or tissue

48.33 Destruction of rectal lesion or tissue by laser

48.34 Destruction of rectal lesion or tissue by cryosurgery

48.35 Local excision of rectal lesion or tissue

48.36 [Endoscopic] polypectomy of rectum

48.4 Pull-through resection of rectum

48.41 Soave submucosal resection of rectum

48.49 Other pull-through resection of rectum

48.5 Abdominoperineal resection of rectum

48.50 Abdominoperineal resection of the rectum, not otherwise specified

48.51 Laparoscopic abdominoperineal resection of the rectum

48.52 Open abdominoperineal resection of the rectum

48.59 Other abdominoperineal resection of the rectum

48.6 Other resection of rectum

48.61 Transsacral rectosigmoidectomy

48.62 Anterior resection of rectum with synchronous colostomy

48.63 Other anterior resection of rectum

48.64 Posterior resection of rectum

48.65 Duhamel resection of rectum

48.69 Other resection of rectum

48.74 Rectorectostomy

48.76 Other proctopexy

48.82 Excision of perirectal tissue

48.99 Other operations on rectum and perirectal tissue

50.22 Partial hepatectomy

50.29 Other destruction of lesion of liver

54.11 Exploratory laparotomy

54.21 Laparoscopy

54.4 Excision or destruction of peritoneal tissue

54.5 Lysis of peritoneal adhesions

54.51 Laparoscopic lysis of peritoneal adhesions

54.59 Other lysis of peritoneal adhesions

54.73 Other repair of peritoneum

54.74 Other repair of omentum

54.75 Other repair of mesentery

68.8 Pelvic evisceration

92.2 Radiotherapy

92.20 Infusion of liquid brachytherapy radioisotope

92.21 Superficial radiation

92.22 Orthovoltage radiation

92.23 Radioisotopic teleradiotherapy

92.24 Teleradiotherapy using photons

92.25 Teleradiotherapy using electrons

92.26 Teleradiotherapy of other particulate radiation

92.27 Implantation or insertion of radioactive elements

92.28 Injection or instillation of radioisotopes

92.29 Other radiotherapeutic procedure

92.3 Stereotactic radiosurgery

92.30 Stereotactic radiosurgery, not otherwise specified

92.31 Single source photon radiosurgery

92.32 Multi-source photon radiosurgery

92.33 Particulate radiosurgery

92.39 Stereotactic radiosurgery, not elsewhere classified

99.25 Injection or infusion of cancer chemotherapeutic substance

99.28 Injection or infusion of biological response modifier [BRM] as an antineoplastic agent

Table 3: Table of the more frequent Diagnosis Related Group codes related to colorectal cancer incidence cohorts 2000-2001.

| **DRG_code** | **DRG_description** | **Cost (Euros)** |
| --- | --- | --- |
| 410 | Chemotherapy without acute leukemia as secondary diagnosis | 1838 |
| 149 | Major small and large bowel procedures without complications | 5138 |
| 148 | Major small and large bowel procedures without complications | 8789 |
| 173 | Digestive malignancy without complications | 3647 |
| 172 | Digestive malignancy without complications | 3944 |
| 147 | Rectal resection without complications | 5836 |
| 181 | Gastrointestinal obstruction without complications | 1793 |
| 189 | Other digestive system diagnoses, age > 17, without complications | 1845 |
| 146 | Rectal resection with complications | 8355 |
| 203 | Malignancy of hepatobiliary sistem or pancreas | 3519 |
| 395 | Red blood cells disorders age >17 | 3394 |
| 409 | Radiotherapy | 2846 |
| 153 | Minor small and large bowel procedures without complications | 3492 |
| 466 | After care without history of malignancy as secondary diagnosis | 1466 |

The table contains the list of the most frequent DRG codes (overall representing about **80**% of all cases) associated to colorectal cancer incidence cohorts 2000-2001. For each DRG [42] description and related reimbursement rate (cost in Euros) are also reported.
